# MicroRNA 320a and Membrane Antigens as Tools to Evaluate the Pathophysiology of Platelets Stored in Blood Banks

**DOI:** 10.3390/cimb44050126

**Published:** 2022-04-22

**Authors:** Priscilla Cristina Moura Vieira, Jersey Heitor da Silva Maués, Letícia Martins Lamarão, Caroline Aquino Moreira-Nunes, Rommel Mário Rodríguez Burbano

**Affiliations:** 1 Human Cytogenetics Laboratory, Biological Science Institute, Federal University of Pará, Belém 66075-110, PA, Brazil; priscillavieira@hotmail.com; 2 Molecular Biology Laboratory, Ophir Loyola Hospital, Belém 66063-240, PA, Brazil; 3 Hematology and Transfusion Medicine Center, University of Campinas, Campinas 13083-970, SP, Brazil; jerseymaues@gmail.com; 4 Foundation Center for Hemotherapy and Hematology of Pará, Belém 66033-000, PA, Brazil; letlamarao@hotmail.com; 5 Pharmacogenetics Laboratory, Drug Research and Development Center, Department of Medicine, Federal University of Ceará, Fortaleza 60430-275, CE, Brazil; 6 Northeast Biotechnology Network (RENORBIO), Itaperi Campus Fortaleza, Ceará State University, Fortaleza 60740-903, CE, Brazil

**Keywords:** miR-127, miR-320a, biomarkers, platelet concentrate, storage lesion, membrane antigens

## Abstract

Our research group, through the analysis of miRNomes in platelet concentrates (PCs) stored in blood banks, identified and validated the miR-127 and miR-320a miRNAs as biomarkers of platelet storage lesions (PSLs) in PCs. In order to validate the miRNAs 127 and 320a methodologically, as PSL biomarkers in a large number of PC bags, we also evaluated important immunological markers involved in the platelet activation/aggregation process—the CD62P receptor (P-selectin), the surface glycoproteins (GP) IIb/IIIa, and the purinergic P2Y12 receptor—via flow cytometry. The miRNAs miR-127 and miR-320a were quantified by real-time quantitative PCR (RT-qPCR). To carry out this study, 500 collection tubes were used at the upper edge of the PC bags containing platelets. Each tube was divided into seven equal parts (totaling 3500 samples) for platelet analysis from 7 different storage days, where the 1st day represents the high-quality control, and the 7th day corresponds to the low-quality control of the platelets. After analyzing all parameters during storage days, it was concluded that the relative quantification of miR-320a below 0.50 and the CD62P receptor below 27.92% are reliable indicators of the absence of storage lesions in blood banks. We believe that the values found in the expression of the CD62P receptor legitimize the use of the miR-320a and miR-127 miRNAs to build a kit capable of accurately measuring whether the stored platelets are suitable for transfusion.

## 1. Introduction

Platelet concentrate (PC) transfusion is widely used in therapeutic transfusion to stop bleeding and maintain stable hemostatic status [1]. This blood component requires special storage; however, even under ideal storage conditions, changes and/or degradation of components in blood bags can occur [2]. These changes, known as platelet storage lesions (PSLs), affect the shelf-life and quality of the stored PCs [3,4].

Storage lesions in PCs include morphological and physiological changes, such as platelet activation, proteolysis, secretion of pro-inflammatory factors, alteration of membrane glycoproteins (GPs), and expression of platelet surface receptors [5,6]. Pathogen-reduction technologies can also affect platelet quality during storage, such as Mirasol treatment, because they decrease platelet function—particularly on the 5th day of storage [7].

Cytometric flow analysis of platelets stored in a blood bank reveals the expression of surface glycoproteins IIb (CD41), IIIa (CD61), and GPIb [8,9], and enables the quantification of P-selectin or 62P differentiation complex (CD62P). CD62P is present in platelets, and is rarely expressed in resting platelets, but can be rapidly released after platelet activation. The presence of platelet surface CD62P is the “gold standard” marker of terminal platelet activation, indicating rupture of the platelet membrane, and the expression of CD62P on the platelet membrane increases during 7-day storage [10]. CD62P is stored in alpha granules of non-activated platelets and Weibel–Palade granules in endothelial cells. After activation begins, with the release of granular content, CD62P starts to be expressed in the platelet membrane, which makes this differentiation complex a good indicator of platelet activation [11]. To the best of our knowledge, there were no studies in the literature that analyzed these four membrane antigens together to try to identify the presence of PSLs in blood banks.

The expression of a large number of platelet miRNAs (total of 219) was first reported by Landry et al. [12] using microarray profiles. In this study, the diverse and abundant existence of miRNAs was demonstrated as a key regulator of the translation of mRNAs in human platelets, establishing the existence and functionality of a process for regulating miRNA-based genes in these anucleate cells. 

Bioinformatics analyzes identified platelet miRNAs (e.g., hsa-miR-320a, hsa-miR-16-5p, hsa-miR-106a-5p, hsa-miR-320b, hsa-miR-15a-5p, hsa-miR-15b-5p, hsa-miR-195-5p, hsa-miR-92a-3p) that modify the expression of proteins associated with platelet activation related to the development of cardiovascular diseases, cerebrovascular diseases, Alzheimer’s, cancer, and hypertension [13].

Studies have suggested that the differential microRNA (miRNA) profile could be a useful tool for identifying changes that occur in platelets during storage [14,15,16], and that platelet physiological changes are related to decreases and increases in the number of miRNAs during the storage period [15,17,18]. 

Reports examining storage conditions have indicated that platelet miRNA profiles vary between different storage times [14,15,17,18,19]. For example, Kannan et al. (2009), when investigating 52 selected platelet miRNAs associated with apoptosis, found that some of these miRNAs were differentially expressed during different stages of platelet storage, where two miRNAs—let-7b and miR-16—showed an increasing profile during storage, while two others—miR-7 and miR-145—showed decreasing profiles, suggesting the use of these miRNAs as potential biomarkers to assess platelet quality during storage [19].

Our research group, through the analysis of a set of expressed miRNAs (miRNome) in the PCs stored in blood banks, identified and validated the miR-127 and miR-320a as biomarkers of PCs’ long-term storage. If the expression of miR-127 is less than that of miR-320a, it indicates that the platelets have undergone in vitro aging and will probably have storage lesions, which would make the PCs unsuitable for transfusion, since this is the main profile observed in PCs from the 4th day of storage. Meanwhile, if the expression of miR-127 is greater than or equal to that of miR-320a, it indicates that the PC bag still has physiologically normal platelets, because this is the profile of the PCs in the first three days of storage [15]. 

Interestingly, in the list of highly expressed miRNAs, we did not find mir-223, which is indicated as a biomarker of the quality of PCs stored in a blood bank [17]; for this reason, we did not include it in the present work. Future studies using mir-223 may provide important information about the role of microRNAs in platelets stored in blood banks, since the expression of mir-223 is associated with the production of the P2Y12 membrane antigen [20].

In order to validate the use of the miRNAs 127 and 320a (miR-127/320a) as PSL biomarkers, in a large number of PCs bags, we also evaluated the quality of the stored platelets by flow cytometry, where we investigated the miRNAs as established markers of platelet activation/aggregation, along with expression of the CD62P receptor and the surface glycoproteins GPIIb and GPIIIa, as well as investigating the role of the P2Y12 receptor, since its function as an ADP receptor in platelet aggregation is not yet fully understood [21].

## 2. Materials and Methods

### 2.1. Collection and Processing of Platelet Samples

To obtain the PCs, a bag of whole blood (ST), or a 450 milliliter (mL) matrix bag, was centrifuged and fractionated using an automatic hematological processor (CompoMat G5 Fresenius Kabi), which separated the platelets from the red blood cells and deposited the PC in a satellite bag, where 99.9% (>3 Log10) of the leukocytes were removed by filtration [22,23]. A PC bag was established by pooling the PCs from five satellite bags taken from healthy volunteers with the same blood type and Rh factor, with negative serological results for blood-borne diseases, provided by the Foundation Center for Hemotherapy and Hematology of Pará (Fundação Centro de Hemoterapia e Hematology of Pará–HEMOPA). 

To assure the presence of only platelets in the tested samples, we performed the leukocyte depletion method as described by Serinolli et al. [24]. PC bags containing 6.0 × 10^10^ platelets each were selected, according to which they were stored under the same conditions used for PC transfusion. The quality control of the PC bags was carried out at the HEMOPA Foundation, which uses the following parameters: swirling, volume, leukocyte count, platelet count, and pH. For this step, on each of the seven days tested, a bag PC tube was subjected to routine quality control tests. All of those parameters were measured by gravimetry as indicated by the National Health Surveillance Agency of Brazil [25]. 

### 2.2. Ethical Statement

This research was approved by the Human Research Ethics Committee of the HEMOPA Foundation for obtaining the PC samples from the ST (consubstantiated opinion no. 194.196) and performed in accordance with the ethical standards as laid down in the 1964 Declaration of Helsinki and its later amendments, or comparable ethical standards. The obtaining of samples did not pose additional risks to the donors, who had signed an informed consent form. 

### 2.3. Experimental Design and miRNA Extraction

To carry out the study, 500 PC bags were used. Each PC bag’s collection tube was divided into 7 equal parts (totaling 3500 samples) to perform the experiments with platelets on 7 different storage days (1st, 2nd, 3rd, 4th, 5th, 6th, and 7th days). That is, on the 1st day of storage, 1/7 of the collection tubes was used to extract miRNAs, and the other 6/7 were stored under the same conditions as the PC bags, at a constant mild temperature of 22 ± 2 °C, to extract the miRNAs on each of the subsequent 6 days. 

According to the criteria of Ordinance No. 158, of 4 February 2016, of the MS of Brazil, which redefines the technical regulation of blood therapy procedures, PC bags must be processed in the first eight hours in circuit, after collecting the ST [25]. For this reason, the first RNA extraction was carried out with 24 h of storage; thus, “newly collected platelets” related to the PCs of the 1st day of storage. As the PCs are stored in a blood bank for a maximum period that varies up to 3 days, depending on the plasticizer of the conservation bag [25], the 1st day represents the high-quality control of the platelets (negative control), and the 7th day corresponds to the low-quality control platelet quality (positive control).

The PC bags used in this study each contained a volume of 3 mL and contained different blood types and Rh factors of the population (of approximately: O+ (55%), O− (4%), B+ (8%), B− (1%), A+ (27%), A− (2.5%), AB+ (2%), AB− (0.5%)).

For miRNA extractions, the mirVana miRNA isolation kit (Thermo Fisher Scientific, Walthan, MA, USA) was used following the protocol suggested by the manufacturer. After extraction, fluorometric quantification of miRNA was performed using a Qubit device (Thermo Fisher Scientific, Walthan, MA, USA). The qualitative assessment of miRNAs was carried out using a NanoDrop (Thermo Fisher Scientific), and the miRNA integrity number (RIN) was determined using the Bioanalyzer 2100 platform (Agilent Technologies, Santa Clara, CA, USA).

### 2.4. miRNA Expression

The real-time quantitative polymerase chain reaction (RT-qPCR) methodology was used to quantify the expression of miRNAs in the collection tube fragments of the PC bags. The mir-191 miRNA was used as an internal control of the efficiency of reverse transcription, and also as a reference miRNA, since it is the most highly expressed on all days of PC storage in the blood bank, as previously described by Pontes et al. [15].

The TaqMan MicroRNA Reverse Transcription kit was used according to the manufacturer’s protocol (Thermo Fisher Scientific, Walthan, MA, USA). The quantification of the expression of the miRNAs miR-127, miR-320a, and miR-191 was measured using the TaqMan PN002229, PN002277, and PN002678 assays, respectively. The complementary DNA was amplified by RT-qPCR using the TaqMan Universal Master Mix II kit with UNG (Thermo Fisher Scientific, Walthan, MA, USA) in the QuantStudio 7 Real-Time PCR Systems thermocycler (Thermo Fisher Scientific, Walthan, MA, USA). All RT-qPCR reactions were performed in triplicate and carried out as described by Pontes et al. [15].

### 2.5. Flow Cytometry

The immunophenotypic platelet study was performed using an Attune NxT Acoustic Focusing Cytometer (Thermo Fisher Scientific) flow cytometer. For immunostaining, the direct immunofluorescence technique was used, where a PC sample of 1.5 × 10^8^ platelets/mL, not fixed in formaldehyde, was pre-incubated for 15 min at room temperature, with five microliters of one of the following monoclonal antibodies: P2Y12 ADP receptor antibody of platelets (Sigma-Aldrich, San Luis, MO, USA), APC conjugate of CD62P/P-selectin antibody (Thermo Fisher Scientific, Walthan, MA, USA), FITC conjugate of integrin antibody alpha-2b/CD41 (Thermo Fisher Scientific, Walthan, MA, USA), or APC conjugate of integrin antibody beta-3/CD61 (Thermo Fisher Scientific, Walthan, MA, USA), which have specificity for the platelet antigens P2Y12, CD62P, GPIIb, and GPIIIa, respectively. The concentration of 5 µL/test was standardized to a 1:200 dilution. Later, the protocol described by Koessler et al. [21] was used.

The characterization of the baseline state of platelet activation—that is, the presence of PSLs in the PCs to be transfused—was performed by evaluating the percentage of platelets positive for CD62P and for GPIIb and -IIIa, as well as by evaluating the intensity of expression of the P2Y12 receptor, as measured by mean fluorescence intensity (MFI). For each PC sample submitted to flow cytometry, 10,000 events were stimulated using the Attune NxT software (Thermo Fisher Scientific, Walthan, MA, USA).

### 2.6. Statistical Analysis

For swirling and PC volume, there was no need to apply statistical analysis. Data from the six biomarkers were tested with Student’s *t*-test at *p* < 0.05 and are presented as means ± standard deviations. The following procedure was adopted: The first day of PC storage was considered the reference value, and the values of the second, third, fourth, fifth, sixth, and seventh days of storage were compared with the first day using Student’s *t*-test (Table 1). Wilcoxon’s paired-samples test was applied to estimate the significance of miR-127 and miR-320a expression in relation to miR-191, which was used as an internal control for validation analysis (*p* < 0.01). In the correlogram analysis, correlation coefficients of miRNAs were calculated with the following functions: cor and rcorr. Correlations with *p*-value > 0.01 were considered to be non-significant. Heatmaps with unsupervised grouping were built with the heatmap.2 function. All statistics and graphs were also constructed using R (https://www.r-project.org, accessed on 14 January 2022).

## 3. Results

### 3.1. Expression of the P2Y12 Purinergic Receptor on the Platelet Surface during Storage of Stored PCs for Different Storage Times

Our results revealed that the baseline level of expression of the P2Y12 receptor on the platelet surface did not show significant variation (Student’s *t*-test), between the storage days (Table 1). 

### 3.2. Expression of the CD62P Receptor and GPIIb (CD41) and -IIIa (CD61) in PCs with Different Conservation Times

**Figure 1:**
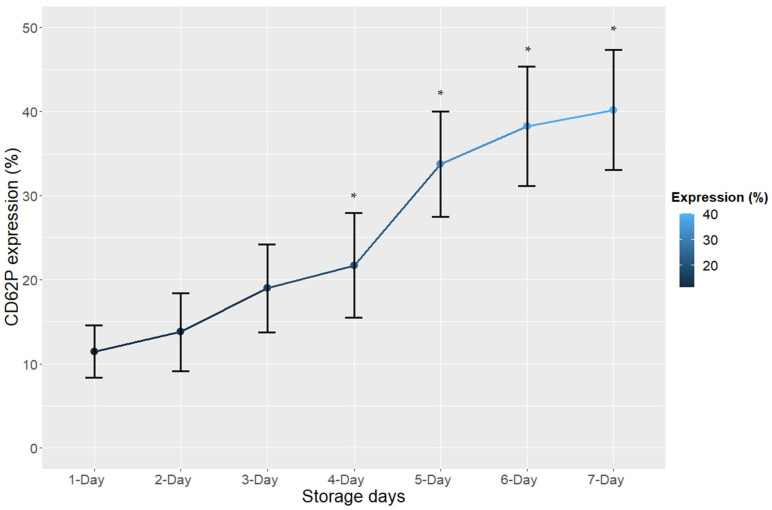
Expression (%) of the CD62P receptor in platelet concentrates during the 7 days of storage. The error bars represent the variability of the data that were calculated from the standard deviation; * *p* < 0.05.

**Figure 2:**
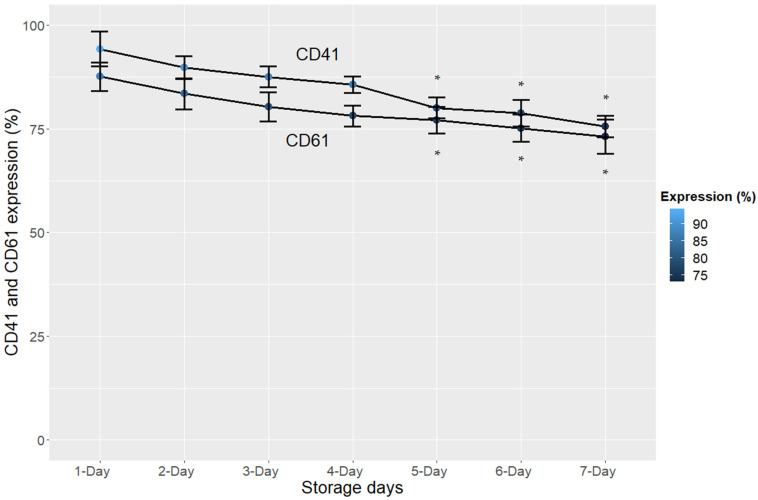
Expression (%) of glycoproteins IIb (CD41) and IIIa (CD61) in platelet concentrates during the 7 days of storage. The error bars represent the variability of the data that were calculated from the standard deviation; * *p* < 0.05.

Regarding the CD62P receptor, our results revealed a significant increase in expression from the fourth day of storage (*p* < 0.05) (Figure 1, Table 1). On the other hand, flow cytometry showed a significant decrease (*p* < 0.05) in the expression levels of glycoproteins IIb and IIIa from the fifth day of storage, which points to the moment of platelet activation or the process of cell damage, as can be seen in Figure 2 and Table 1.

### 3.3. Expression of miR-127 and miR-320a miRNAs in PCs with Different Conservation Times

The miR-127 miRNA showed a higher level of expression quantification than miR-320a during the first three days of storage, and in the following three days these miRNAs changed their position. This phenomenon was observed in all 3500 samples studied, without exception. However, there was no significant difference in miR-127 variation. On the other hand, on the third day of miR-320a storage, there was a significant increase in the relative quantification of this miRNA (*p* < 0.05).

**Figure 3:**
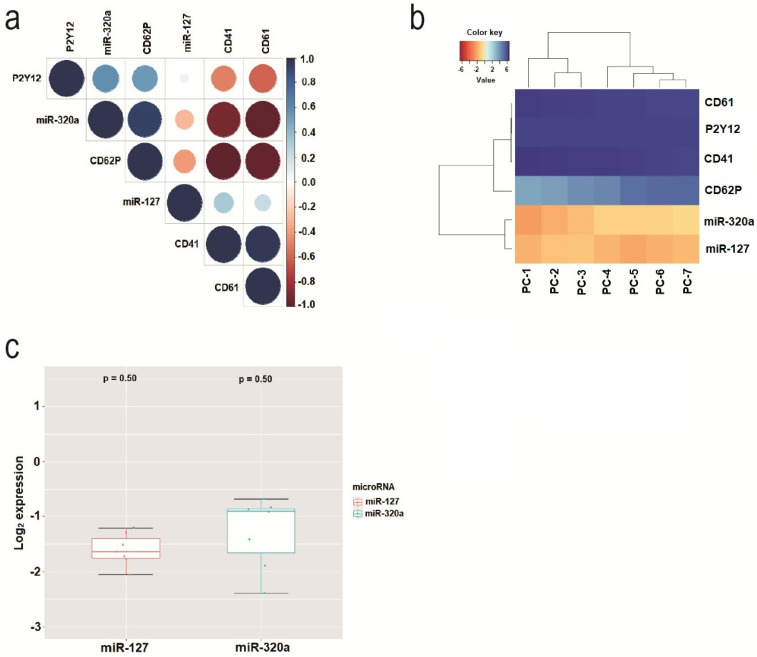
(**a**) Correlogram showing the miRNAs and membrane receptors that most correlated during the 7 days of PC storage. The scales with a blue gradient are positively correlated, while those with a red gradient are negatively correlated; the white gradient represents insignificant correlation (*p* > 0.01). (**b**) Heatmap showing the patterns of expression of miRNAs and receptors during the 7 days of storage of PCs. Z-score was the metric applied to infer the best clustering patterns. Gradients with a tendency towards a red color indicate a lower Z-score, and gradients with a tendency towards a blue color indicate a greater Z-score. (**c**) Variation in the levels of expression of miR-127 and miR-320a during the 7 days of storage.

Figure 3a shows a correlogram calculated to highlight the miRNAs and membrane receptors that most correlated during PC storage. miR-320a showed a higher correlation than miR-127. The membrane receptors that showed the highest correlation were P2Y12 and CD62P. Figure 3b shows a heatmap showing the expression patterns of miRNAs and receptors during the seven consecutive days of storage. It was observed that CD62P was grouped by the expression level closest to the expression level of miR-320a. Figure 3c shows the variation in miR-127 and miR-320a expression levels during the 7 days of storage. The expression was normalized to the Log2 scale.

## 4. Discussion

Studies have reported that the majority of changes in storage lesions of PCs are related to platelet activation [26,27,28].Thus, this study investigated important immunological markers involved in the platelet activation/aggregation process—the CD62P receptor, the GPs GPIIb and GPIIIa, and the ADP receptor, (P2Y12)—via the traditional methodology of flow cytometry. In addition, this study also evaluated the parameters swirling, volume, leukocyte count, platelet count, and pH, which are already used by the HEMOPA Foundation’s quality control.

In fact, GPIIb and GPIIIa are more important in mediating platelet aggregation than in inflammation [29]. In the present work, the objective of monitoring the levels of GPIIb and GPIIIa was to observe whether from the fourth/fifth day—when the storage lesions appeared—the levels of GPIIb and GPIIIa expression increased, which in fact happened on the fifth day of the storage period, confirming their role in platelet aggregation. We believe that GPIIb and/or GPIIIa are late markers of platelet activation or cell damage, since they only showed a significant increase (*p* < 0.05) on the fifth day of platelet storage.

Our results revealed an increase in the levels of CD62P quantification (*p* < 0.05) on the platelet membranes during the fourth day of storage (Figure 1, Table 1). We hypothesized that the increase in this quantification corresponds to a later biological phenomenon, since it is a protein, compared to the quantification of miR-320a, which presented a more sensitive biological reaction to aging or platelet activation in the blood bank on the third day of storage (*p* < 0.05) [15]. For this reason, the platelet response in the blood bank to storage lesions can be sensitively assessed using the CD62P antigen and miR320a. CD62P expression on the platelet surface is considered an important indicator of platelet activation [11,30], and most previous studies indicate that increased CD62P expression in platelets is associated with storage injuries [31,32]. Therefore, the results of this study corroborate the literature, and confirm the possibility of using this receptor as a biomarker of storage lesions in PCs. According to our results, when the expression of this receptor in the platelet membrane does not exceed 27.92%, it is an indication that the platelets are in good storage conditions, since on the fourth day of storage the average expression of the CD62P membrane was 21.70%, and the variation in 500 PC bags was ±6.22 (Table 1), which together adds up to 27.92%.

The analysis of the immunological marker P-selectin and the glycoproteins GPIIb/IIIa, obtained via flow cytometry, cannot be used as a definitive criterion for platelet pre-activation. The externalization of P-selectin is accompanied by the loss of mitochondrial membrane potential, and its expression variation—together with that of glycoproteins—may be related to other platelet functional responses that may also influence the evaluation of the quality and degree of deterioration of P-selectin in stored platelets [7].

As for the expression of glycoproteins IIb (CD41) and IIIa (CD61), our results revealed a decrease only on the fifth day of storage (*p* < 0.05) via flow cytometry, which points to the moment of platelet activation or cell damage (Figure 2), and for this reason, according to the results of this study, we cannot use them as biomarkers of PC quality. Nasiri and Vaeli [33] also described a late degradation of these glycoproteins during the storage of platelet concentrates, which corroborates the data presented in this work.

Studies show that ADP signaling and ADP-induced aggregation/activation are affected in stored platelets [34,35], which is why we decided to study the P2Y12 receptor. The flow cytometric analysis of P2Y12 receptor expression did not reveal variation in its baseline levels during the storage days. Our results corroborate those found in the study by Koessler et al. [21], who investigated the activation of both membrane surface P2Y12 receptors and receptors present within platelets that are released during platelet activation, and also found that the expression values of this receptor remained unchanged during storage. The P2Y12 receptor is the target of antiplatelet drugs used in the treatment of patients with cardiovascular diseases and implanted coronary stents, and its inhibition is associated with an anti-aggregating effect and hemorrhagic diathesis [36,37]. Thus, the preserved activity of the P2Y12 receptor in stored platelets may be an important requirement for the physiological functionality of platelets used in transfusions [21]. On the other hand, it is possible that the types of storage lesions that platelets suffer during storage are not sufficient for the receptor to be activated by the ADP. Future studies must be carried out in order to clarify this issue.

Regarding the quality control parameters, the results were shown to be compatible with the immunological markers. The presence of swirling was observed up to the fifth day of storage, indicating a change in platelet shape from the sixth day. Swirling is an index that is used to indirectly assess the quality of platelets; it is normal, and there is a consensus that due to storage lesions on the sixth day it does not appear, because there is a change in the discoid shape of the platelets, also demonstrating the need to discard PCs [28]. For this reason, we propose more indices of PC assessment to be sure of the poor quality of the platelets before they are discarded.

The volume of PC used for extraction (3 mL) remained constant, which shows that it is airtight and suitable for blood banking. The leukocyte count decreased significantly from the fifth day of storage (*p* < 0.05), probably due to their degradation, which promotes the release of inflammatory immunomodulators, which may lead to platelet activation [38]. Therefore, leukoreduction (leukocyte filtration) is an important procedure for better conservation of PCs stored in blood banks. The platelet count did not decrease significantly throughout the storage period; thus, the values found on all seven days of storage are in accordance with the minimum levels recommended by Brazilian technical standards [25]. The pH remained constant until the third day, and increased from the fourth day onwards, which may be indicative of an increase in cellular metabolism, showing a direct relationship with the beginning of the platelet activation phenomenon, concomitant with the increase in the quantification of CD62P and miR-320a expression.

Due to the occurrence of PSLs, blood banks dispose of unused PC bags after five days of storage, and on the sixth day there may be a shortage of PCs in the stock, as a result of which blood transfusion services face a serious problem affecting public and private health—the chronic shortage of platelets [39]. Although platelets are collected daily in Brazil—mainly in the north of the country—the number of volunteers is insufficient, and the demand is greater than the collection. Additionally, there is a priority for the use of platelets with a few days of storage, due to the lack of quality tests such as the one we propose here for this reason. However, many of these PC bags may still contain functional platelets and be viable for transfusion, since the kinetics of cell aging are influenced by biological and environmental factors, which are inherent to each blood donor [40,41]. For this reason, the disclosure of tools that can better identify the occurrence of PSL and, therefore, the quality of the stored blood products, is of great interest to the transfusion medicine community [42]. 

The co-expression profiles of miRNA–mRNA were correlated with platelet reactivity, consolidating the importance of the regulatory role of miRNA in modulating platelet mRNA translation. As platelets suffer storage damage or are activated by the presence of microorganisms, there is an initial process of miRNA degradation that reduces their quantification. However, this process ceases, and some miRNAs may have their quantification increased as cell damage or infection evolves, since RNA-editing enzymes—such as RNase and RNA helicases—increase the quantification of certain miRNAs through cleavage of miRNA precursors (pre-miRNAs) in mature miRNAs, in response to oxidative stress [15,16,17,43,44].

This hypothesis could explain the increase in the quantification of miR-320a in relation to miR-127 (Table 1), since precursors of miR-320a can be cleaved to produce a greater number of this miRNA, as miR-320a has a regulatory capacity to inhibit protein translation in response to stress [45]. In this case, specifically, the stress would be due to the process of the appearance of storage lesions. Evidence that supports our hypothesis is that miR-320a is responsive to oxidative stress and can regulate glycolysis in clinical diseases that are associated with changes in energy supply. In this case, the level of miR-320a increases up to 100-fold due to cleavage of the pre-miR-320a [46].

In addition, it has also been shown that musculoskeletal pain conditions increase circulating levels of miR-320a, indicating that this miRNA is responsive to stress [47]. Cell culture analyses indicate that miR-320a expression is also modulated by cell stress [46]. Hyperoxia also increases the levels of miR-320a in microvesicles derived from epithelial cells [48]. However, the most important evidence comes from Nikulin et al. [49] who found, in Caco-2 cell culture under the conditions of microcirculation in a microfluidic device enabling them to simulate the natural environment of the body, that the secretion of miR-320a is indicative of cell stress and activation of the inflammatory response.

To the best of our knowledge, there is no documented association of inflammatory or non-inflammatory oxidative stress with miR-127 in the literature. However, in the future, other miRNA reference controls, in addition to miR-191, need to be tested. We used miR-191, as this was the most highly expressed on all six days of PC storage. The list of highly expressed miRNAs was slightly different from previous studies—mainly miR-223 [44,50,51]—probably because the sequencing analyses that were performed on samples stored in a blood bank, where the first RNA extraction was performed after 24 h of storage, may have induced a slight variation.

As expected, the analysis of the miRNAs miR-127 and miR-320a revealed greater quantification of miR-127 compared to miR-320a during the first three days of storage, and in the following three days these miRNAs changed positions. This phenomenon was observed in the 500 PCs, and it probably occurs because miR-127 degrades more quickly than miR-320a. This inverse expression relationship between these miRNAs, depending on the storage time, can allow us to identify PC bags that, even at an advanced storage period (4th and 5th days), still present physiologically normal platelets that could, in the event of the absence of fresher PCs, be used in a blood transfusion. We found that the relative quantification of miR-320a below 0.50 is a reliable indicator that platelets do not yet show storage lesions in blood banks, since on the third day of storage the average relative expression of mir320 was 0.3,7 and the maximum variation in 500 PC bags was ±0.13 (Table 1, Figure 3c), which would add up to 0.50. This value would be within the confidence limits, since they would not reach the values of 0.54 and 0.56 of the fifth and sixth day of storage, respectively, of this microRNA in platelets stored in a blood bank.

We must consider that antibodies to purinergic receptors can be very nonspecific, and flow cytometry may not be the most suitable method to detect the P2Y12 receptor. To solve this problem, as mentioned in the Materials and Methods, for all biomarkers analyzed in this study, we used PCs from the first day of storage as a negative control, where the concentration of P2Y12 in the membrane of the platelets is expected to be low, and as a positive control we used PCs from the seventh day of storage, where the concentration of P2Y12 is expected to be high. In fact, there was an increase in the concentration of P2Y12 as the storage days increased, until the seventh day, but it was not enough to find a statistically significant difference, as in the other membrane antigens. For the measurement of P2Y12, an alternative would be to study the platelet response to ADP using functional aggregation tests, but this was a limitation of our study that was not predicted at the time of execution. This study presents the prospect of carrying out a proof-of-concept study to confirm the relevance of using the biomarkers discussed here to qualify a PC bag, via in vitro functional tests, in PC bags stored for more than 7 days.

Despite improvements in donor screening and increased efforts to avoid contamination and the spread of pathogens in platelet concentrates (PCs), our study evaluated platelets stored in a blood bank without the use of pathogen-reduction technologies (PRTs). However, the risks of transfusion-transmitted infections remain important. Based on an ultraviolet photoactivation system, PRT technologies measure the inactivation of pathogenic nucleic acids. After activation, platelets release microparticles (MPs), which are involved in intercellular communications and the regulation of gene expression, thus mediating critical cellular functions. Considering that platelet MPs can transfer their miRNA content to recipient cells, and that this content can exert biological activities, these findings suggest that the PRT treatment of clinical PCs can modify the bioactivity of the platelets and MPs to be transfused, and so the authors argue for the need for further investigation into PRT-induced changes in CP content and clinical function [52].

Platelet storage lesions are widely reported in the literature. The use of platelet membrane antigens and other criteria described in the table has already been reported in the literature [53,54]. Our research group recently performed a combined analysis of miR-127 and miR-320a expression and membrane antigens in platelets, in association with classical clinical and laboratory parameters, and demonstrated that this is an important tool to detect the onset of sepsis [55], which corroborates the results presented in this work to detect the beginning of the deterioration of platelets stored in a blood bank.

In the literature, and in clinical practice, the biomarkers presented here, when analyzed separately, have limited scientific value in hemotherapy. This study is a pioneer in performing a combined analysis of the quantification of microRNA and cellular receptors, which is a field that still needs to be better elucidated. However, the data presented in this work may contribute to a better understanding of the complexity of PC storage injuries.

## 5. Conclusions

In conclusion, after analyzing all PC parameters, we can conclude that the relative quantification of miR-320a below 0.50 and the CD62P receptor below 27.92% are reliable indicators of the absence of storage lesions in blood banks. We believe that the values found in the expression of the CD62P receptor legitimize the use of the miR-320a and miR-127 miRNAs to build a kit capable of accurately measuring whether the stored platelets are suitable for transfusion.

## Figures and Tables

**Table 1:** Parameters evaluated in platelet concentrates.

Parameters	Day 1	Day 2	Day 3	Day 4	Day 5	Day 6	Day 7
* Quality control							
Swirling	Positive	Positive	Positive	Positive	Positive	Negative	Negative
PC volume	3 mL	3 mL	3 mL	3 mL	3 mL	3 mL	3 mL
Leukocyte count (10^8^/70 mL)	0.51 ± 0.02	0.48 ± 0.02	0.42 ± 0.03	0.34 ± 0.03	0.25 ± 0.04 *	0.18 ± 0.06 *	0.12 ± 0.06 *
Platelet count (10^10^)	7.3 ± 0.10	6.9 ± 0.11	6.7 ± 0.15	6.4 ± 0.18	6.1 ± 0.21	5.8 ± 0.24	5.5 ± 0.30
pH	7.2 ± 0.02	7.2 ± 0.03	7.2 ± 0.01	7.3 ± 0.01	7.3 ± 0.02	7.3 ± 0.01	7.4 ± 0.05
miRNAs expression **							
miR-127	0.30 ± 0.12	0.41 ± 0.16	0.43 ± 0.19	0.32 ± 0.17	0.24 ± 0.23	0.29 ± 0.21	0.35 ± 0.25
miR-320a	0.19 ± 0.11	0.27 ± 0.13	0.37 ± 0.13 *	0.53 ± 0.18 *	0.54 ± 0.19 *	0.56 ± 0.22 *	0.62 ± 0.22 *
**Membrane proteins’ expression +**							
P2Y12	39.57 ± 2.14	39.48 ± 1.73	40.56 ± 1.97	41.64 ± 1.56	41.19 ± 1.28	42.87 ± 1.80	42.69 ± 1.32
CD62P	11.45 ± 3.12	13.76 ± 4.61	18.96 ± 5.22	21.70 ± 6.22 *	33.76 ± 6.25 *	38.26 ± 7.11 *	40.20 ± 7.17 *
CD41	94.32 ± 4.20	89.81 ± 2.81	87.54 ± 2.52	85.73 ± 1.98	80.11 ± 2.53 *	78.81 ± 3.22 *	75.54 ± 2.60 *
CD61	87.62 ± 3.43	83.51 ± 3.72	80.31 ± 3.53	78.13 ± 2.46	77.14 ± 3.25 *	75.21 ± 3.27 *	73.11 ± 4.10 *

Values are expressed as means ± standard deviations. * Gravimetric measurement. ** Values referring to the relative quantification of miRNAs mir127 mir320a, for mir-191. + Percentage of platelets positive for CD62P and for GPIIb and -IIIa. For the P2Y12 receptor, the mean fluorescence intensity (MFI) was evaluated.
